# Self-healing hybrid hydrogels with sustained bioactive components release for guided bone regeneration

**DOI:** 10.1186/s12951-023-01811-8

**Published:** 2023-02-22

**Authors:** Jiaxin Li, Weichang Li, Mengjie Kong, Zongtai Li, Tao Yang, Qinmei Wang, Wei Teng

**Affiliations:** 1 Hospital of Stomatology, Sun Yat-sen University, Guangzhou, 510055 People’s Republic of China; 2 Guangdong Provincial Key Laboratory of Stomatology, Guangzhou, 510055 People’s Republic of China; 3 Guanghua School of Stomatology, Sun Yat-sen University, Guangzhou, Guangdong People’s Republic of China; 4 Laboratory of Biomaterials, Key Laboratory on Assisted Circulation, Ministry of Health, Cardiovascular Division, First Affiliated Hospital, Sun Yat-sen University, Guangzhou, People’s Republic of China

**Keywords:** Self-healing, Hybird hydrogel, Bioactive components, Barrier membrane, Guided bone regeneration

## Abstract

Guided bone regeneration (GBR) is widely used in treating oral bone defects to exclude the influence of non-osteogenic tissue on the bone healing process. The traditional method of GBR with a titanium mesh to treat large-area bone defects is limited by the deficiency of increased trauma and costs to patients. Herein, a bi-layered scaffold for GBR composed of a fiber barrier layer and a self-healing hydrogel repair layer is successfully fabricated. The barrier layer is a fibrous membrane material with specific porosity constructed by electrospinning, while the functional layer is a self-healing hydrogel material formed by multiple dynamic covalent bonds. The system can provide an osteogenic microenvironment by preventing the infiltration of connective tissue to bone defects, maintain the stability of the osteogenic space through the self-healing property, and regulate the release of bioactive substances in the dynamic physical condition, which is beneficial to osteoblast proliferation, differentiation, and bone regeneration. This study focused on exploring the effects of different crosslinkers and bonding methods on the comprehensive properties of hydrogels. and proved that the hybrid scaffold system has good biocompatibility, cell barrier function and can enhance bone regeneration activity. Thereby it could be a promising clinical strategy for bone regeneration.

**Supplementary Information:**

The online version contains supplementary material available at 10.1186/s12951-023-01811-8.

## Introduction

The main challenge of traditional dental implants and denture repair is bone tissue damage and defects caused by fractures, age, infection, tumors, and genetic diseases [1–3]. Once exceeding the self-repairing capacity of bone tissue, external interventions are required to accelerate bone regeneration to recover normal bone function [4–6]. In oral implantology, guided bone regeneration (GBR) is a surgical approach that prevents the migration of fast-moving epithelial cells and fibroblasts into bone defects, which is suitable for proliferation, differentiation, and bone regeneration of osteoblasts as an effective and simple method for bone augmentation to rebuild alveolar bone [7]. GBR could also be used in large bone defects titanium mesh [8]. However, the patient must undergo a second operation to remove the titanium mesh. In contrast, recent studies have focused more on degradable self-healing hydrogel scaffolds, which have great biocompatibility, and maintain osteogenic space stability. We propose that this feature may make them suitable for bone tissue engineering. Self-healing capabilities in hydrogels are chemically or compositionally incorporated directly into the polymer structure via the incorporation of reversible bonds (crosslinks/reactions) [9]. Hence, we propose the use of a hybrid hydrogel (PVGM) by interpenetrating polymer networks of methacrylated gelatin (GelMA) and polyvinyl alcohol (PVA). Then we introduce 3,4-dihydroxybenzaldehyde (DBA) and 4-vinylphenylboronic acid (PBA) into PVGM, respectivly. These materials are nontoxic, biocompatible, biodegradable, and has been used in many biomedical products [10–14]. On the other hand, the vinyl group of PBA can be connected to the hydrogel network (PVGM/P) by a dynamic boronic ester bond, and the aldehyde group of DBA bonding the hydrogel (PVGM/D) through hydrogen bonds. Meanwhile, the reversible hydrogen bonds endow the system with self-healing properties. To the best of our knowledge, there is no published report discussing the effects of different bonding methods on the comprehensive properties of hydrogels.

Bioactve substances carried by hydrogels profound influence on biological functions. Recent studies suggested that DNA nanostructures have advantages of excellent editability and biocompatibility for biomedical applications. However, there are exogenous DNA, yield, stability, and ethical considerations regarding their clinical translation [15–17]. Biological and synthetic organic-inorganic composites have been explored. Nano-hydroxyapatite (nHA) showed better biocompatibility, excellent bioactivity, osseointegration ability, and less inflammatory response [18–21], and improved the osteogenic properties of the hydrogel even toughening it [22]. Hence, we added nHA into the hydrogel scaffold (PVGM/D@nHA).

**Fig. 1:**
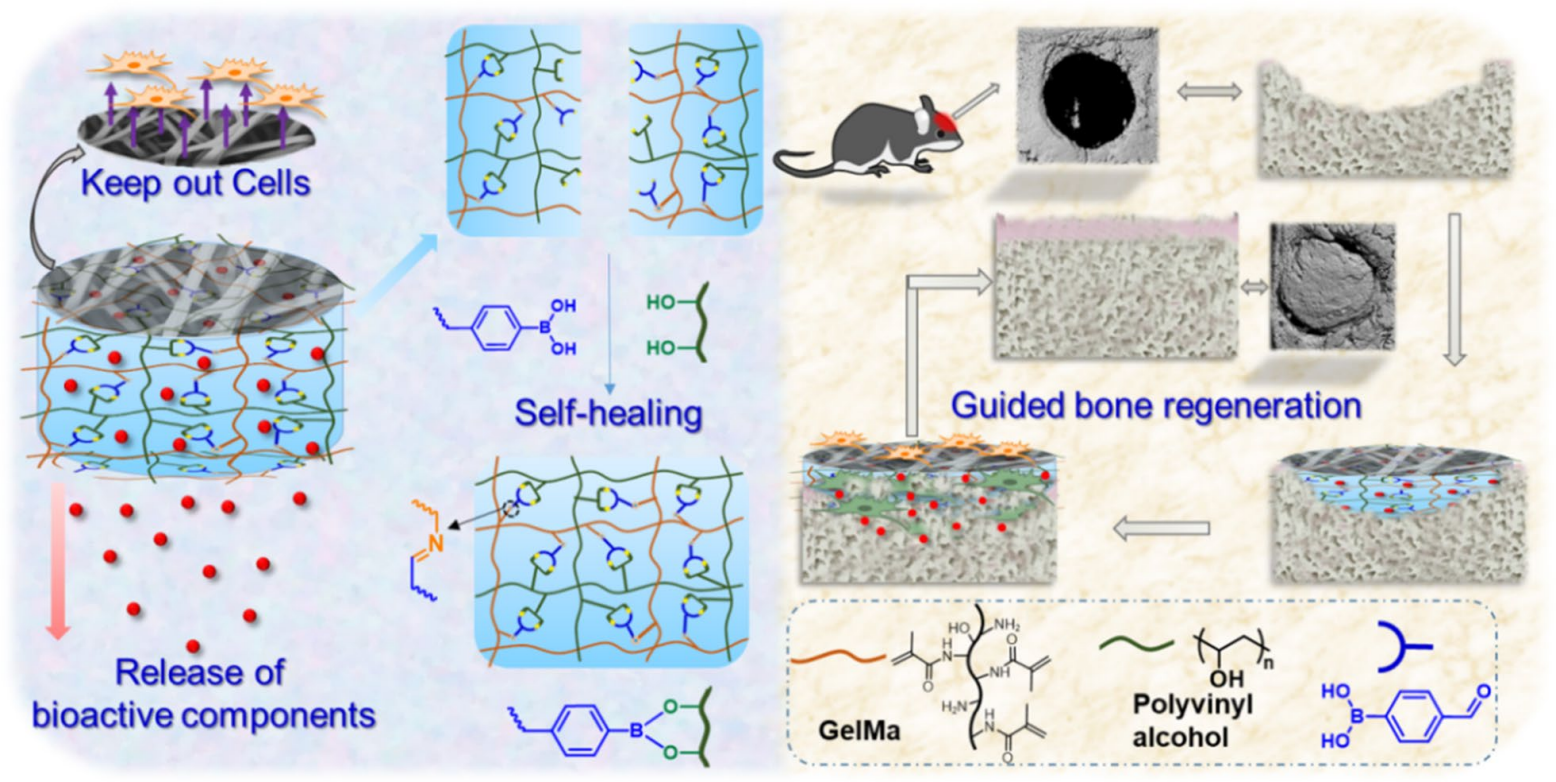
Schematic illustration of the preparation and application of hybrid scaffold system. The hydrophobic layer was prepared by electrospinning technology, and the hydrogel scaffold formed by cross-linking; the obtained composite scaffold was capable of fibroblasts barrier, self-healing, calcium ions release, and bone regeneration

PLGA carrying amino groups (PLGA-NH2) were bound to the surface of the hydrogel (PLGA_PVGM/D@nHA) rich in catechol structure by electrospinning technology acting as GBR membrane (Fig. 1). In this work, a series of hydrogel scaffolds with different concentration gradients of nHA were prepared, and their mechanical properties and osteogenesis effect in vitro of BMSCs were evaluated. Therefore, the ratio of nHA in the hydrogel was optimized. The mineralization properties of the hydrogel scaffolds are determined in vitro. We will focus on evaluating the ability of the PLGA_PVGM/D@nHA hydrogel scaffold to recruit natural cells and improve bone formation in a critical-size rat calvarial defect model. This bi-layered hydrogel scaffold may serve as an integrative bone graft device with multifunctional components for clinical bone repair.

## Materials and methods

### Materials

Gelatin from chicken bones (type B, reagent grade, Aladdin), methacrylic anhydride (94%, Aladdin, China), polyvinyl alcohol (98%, Sigma-Aldrich, St. Louis, USA), 3,4-dihydroxybenzaldehyde (98%, J & K Scientific, China), 4-vinylphenylboronic acid (98%, J&K Scientific, China), PLGA-NH2 (Mn 100000, Chongqing Yusi Pharmaceutical Technology Co., Ltd, China), nano-hydroxyapatite (99%, J & K Scientific, China). Sodium hydroxide (98%, Sigma-Aldrich, St. Louis, USA), sodium bicarbonate (99.8%, Aladdin, China), 2-hydroxy-4’-(2-hydroxyethoxy)-2-methylpropiophenone (IRGACURE 2959, 99%, Adamas-beta, China), ammonium persulfate (APS; 99.9%, Aladdin, China), 6,6'-bisamino-3,3'-methylidene dibenzoic acid (MBAA; 99%, Aladdin, China), and dichloromethane (99.9%, Macklin, Shanghai, China) were used as received.

### Synthesis of methacrylated gelatin (GelMA)

50 g type B chicken bone gelatin was dissolved in 1000 ml of deionized water at 45 °C with stirring. The solution pH was adjusted to 7.4 by dropwise addition of sodium hydroxide and stirring until it became homogeneous and transparent. Then, 40 ml methacrylic anhydride was slowly added at a speed of 0.5 mL min^−1^, and the reaction proceeded at 45 °C for 2.5 h. The mixture was precipitated with ultracentrifuge, and the collected supernatant was dialyzed against deionized water. The molecular weight cut-off (MWCO) of dialysis was 3500, and the dialysis time lasted 7 days. Finally, the purified solution was lyophilized to obtain GelMA.

### Preparation of PVGM, PVGM/D, PVGM/P, PVGM/D@nHA and PLGA_PVGM/D@nHA hydrogels

PVGM copolymer hydrogels were prepared by photo-initiated radical copolymerization. Briefly, an appropriate mass of PVA and GelMA was first dissolved in deionized water to prepare a 5 wt% GelMA solution and a 10 wt% PVA solution, respectively. Then 1 wt% photoinitiator IRGACURE 2959, 1 wt% APS and 1 wt% MBAA (relative to the total weight of monomers) were added to the solution. 4 wt% DBA or PBA powder was added into PVGM hydrogel solution to obtain PVGM/D or PVGM/P hydrogel solution. Similarly, for PVGM/D@nHA hydrogel, nHA was dissolved in 500 μl deionized water according to the designed formulations, then a 200 μl mixture was added to 5 ml of PVGM/D hydrogel solution. In this study, a series of hydrogels were prepared by the varying initial concentration of nHA. To simplify the discussion, the obtained hydrogels were coded as PVGM/D@XnHA, where X represented the initial mass percentage concentration of nHA. Subsequently, these mixtures were cast into plastic rectangular molds and polymerized in a crosslink oven (Scientz03-IIUV CrossLinker, Ningbo Scientz Biotechnology, China) for 60 min.

For PLGA_PVGM/D and PLGA_PVGM/D@nHA hydrogel, electrostatic spinning was applied to fabricate a PLGA fiber layer. 20 wt% PLGA was dispersed in DCM stirring until complete dissolution. Then, the PLGA membrane formed by electrostatic spinning was flattened into a plastic rectangular mold injected with PVGM/D or PVGM/D@nHA hydrogel solution. Then, PLGA_PVGM/D and PLGA_PVGM/D@nHA hydrogel were formed by UV light irradiated polymerization.

### Characterizations of hydrogels

#### Surface morphology

The surface topography and elements of the hydrogels were observed by scanning electron microscopy (SEM) and energy disperse spectroscopy (EDS). After lyophilization of all hydrogels, the surfaces were sputter-coated with gold layers and then analyzed using the Thermal Field Emission Environmental SemEdsEBSD (FEI Quanta 400 F, Netherlands). At least 30 pores and 30 fibers in the obtained SEM images were evaluated using Image J software to analyze the mean pore and fiber diameters.

A laser scanning confocal microscope profiler (LSM700, Zeiss, Germany) was used to observe the surface roughness of the membrane and hydrogel. Two roughness parameters were investigated: Sz (the maximum height) and Sa (the arithmetic mean height deviation from the mean plane).

JCY-1 contact angle system (Shanghai Fangrui Instrument Co., Ltd., Shanghai, China) measured water contact angles**.**

#### FTIR spectroscopy

Fourier transforms infrared (FTIR) spectroscopy was used to characterize the formation of PVGM, PVGM/D, PVGM/P hydrogels, the successful doping of nHA into the PVGM/D hydrogels, and the bonding between PLGA membrane and hydrogels.

#### Dynamic swelling behaviors and equilibrium water contents (EWCs) of PVGM, PVGM/D, PVGM/P, and PVGM/D@nHA hydrogels

To observe the dynamic swelling behaviors, the fresh gels were weighed on a microbalance to record their initial wet and dry weights (Wi), and then immersed in deionized water at 37 °C, separately. The weight of the hydrogel in the swollen state (Wt) was quickly recorded after gently blotting out excess water from the surface. The swelling ratio of the hydrogels can be calculated by the Eq. (1):

1 $$Swelling\,Ratio\left(\%\right)= \frac{{W}_{t}}{{W}_{i}}\times 100\%$$

where Wt is the wet weight of the swollen hydrogel at time t (t is the time that the hydrogel was soaked in deionized water), and Wi is the initial wet weight (or dry weight) of the hydrogel. The equilibrium water contents (EWCs) of the hydrogels were determined according to the reported method [23].

#### Mineralization properties of PLGA_PVGM/D@nHA Hydrogels

The apatite forming characteristics of the hydrogels in vitro were tested at 7.4 pH by immersing 100 mg hydrogel into 1 ml simulated body fluid (SBF). The mineralization morphology was observed by SEM (FEI Quant400F, The Netherlands). An inductively coupled plasma-atomic emission spectrometer (ICP-AES, TJA, USA) was used to detect the release of calcium (Ca) and phosphorus (P) ions in the solutions.

#### In vitro* degradation of PLGA_PVGM/D@nHA hydrogels*

The degradation behavior of different hydrogels was determined by the enzymatic degradation process. The circular samples were immersed in phosphate buffer (PBS) at 37 °C, added with freshly prepared collagenase II solution (25 U/mL) [24], and were then removed at predetermined time intervals, rinsed thoroughly with deionized water, then lyophilized and weighed. The remaining mass percentage was calculated according to Eq. (2) [21]:

2 $$\mathrm{Remaining\,Weight }\left(\%\right)=\frac{{W}_{0}-{W}_{t}}{{W}_{0}}\times 100\%$$

where W_0_ is the initial weight of the sample and Wt of each incubated time point.

#### Mechanical properties of PVGM, PVGM/D, PVGM/P, and PVGM/D@nHA hydrogel scaffolds

The mechanical tests were performed using a universal testing machine (Instron 5967, USA) at room temperature. Hydrogels were extended at a rate of 1 mm min^−1^ until failure at tensile test and were compressed to 40% strain at a deformation rate of 1 mm min^−1^ at compression tests. Cyclic measurements were performed 3 times with the same speed of 1 mm min^−1^ [25].

#### Self-healing properties

The hydrogel samples (30 mm in diameter, 4 mm in height) were cut into two pieces with a knife, then simply butted and put into the mold. The self-healing process was carried out in a 45 °C water bath under sealed conditions, then recorded with a digital camera after 48 h.

#### Protein adsorption and cell attachment, viability, and proliferation

The hydrogels (5 mm × 5 mm × 2 mm) were placed in 48-well plates and immersed in bovine serum albumin (BSA) solution (2 mg/mL). After 6 and 24 h of incubation, the BCA Protein Analysis Kit (Nanjing Jiancheng, China) was conducted according to the instructions.

After the BMSCs (1 × 10^4^ cells per well) were seeded on the hydrogels, the hydrogels were stained with Actin Tracker (Beyotime, Shanghai, China) and DAPI (Beyotime, Shanghai, China) or the Calcein-AM/PI Double Stain Kit (Best BioScience, Nanjing, China) to evaluate the cell viability. The laser scanning confocal microscope 780 (Zeiss, Oberköchen, Germany) was then used to observe these cells.

Cell proliferation was evaluated using the Cell Counting Kit (CCK-8, Dojindo, Japan). The microplate reader (Thermo 3001, USA) was used to measure the optical density (OD) at 450 nm.

#### Cell infiltration measurement

The L929 cells were seeded onto PLGA membranes for cell permeation experiments. The samples were then fixed into the transwell chamber (Bayton, Dickinson, NJ, USA) and placed into a 24-well plate, while the control group was fixed without any samples. The samples fixed in the chambers, the 24-well plate, and the bottom of the control chambers was stained with Hoechst 33342 (Beyotime, Shanghai, China). Cell infiltration behavior was observed using a laser scanning confocal microscope 780 (Zeiss, Oberkochen, Germany).

#### Osteogenic differentiation evaluation

BMSCs were seeded in a 48-well plate at a seeding density of 1 × 10^4^ cells per well. The extracted medium was prepared by immersing the sterilized PVGM/D@nHA in an osteogenic induction medium (standard medium supplemented with 10 nM dexamethasone, 10 mM β-glycerol phosphate, and 50 μg/mL of L-ascorbic acid) at 37 °C for 24 h. The extraction ratio of PVGM/D@nHA hydrogel to culture media was 0.2 g/ml.

Alkaline phosphatase (ALP) expression on the 4th and 7th day was quantified with Alkaline Phosphatase assay Kit (Beyotime, China) and observed by BCIP/NBT Alkaline Phosphatase Color Development Kit (Beyotime, China), which the hydrogel was observed with a fluorescence stereo microscope (Lecia, MZ10 F).

To evaluate the mineralized extracellular matrix, the BMSCs were immersed in 1% Alizarin Red S (ARS) solution for 5 min, then the stained cells were observed with an OLYMPUS SZX16 stereo microscope.

The expression of osteogenic genes was detected by quantitative polymerase chain reaction (qPCR). RNA-Quick Purification Kit (Yishan Biotech, Shanghai, China) was used to extract the total cellular RNA. Then, RNA was reverse transcribed into cDNA by PrimeScript^™^ RT reagent Kit (TaKaRa, Japan). The expression of related genes standardized by the housekeeping gene, reduced glyceraldehyde phosphate dehydrogenase (GAPDH), was detected by a real-time qPCR system (ABI QuantStudio 5, USA). The expression of related genes was compared using the 2^−ΔΔCt^ method. The primer sequences used in this study were shown in Additional file 1: Table S4 (Additional file 1).

Immunofluorescence staining was used to observe the expression of Runx2 and Opn protein in BMSCs. Cells were fixed and incubated with Anti-Runx2 antibody (#12556, Cell Signaling Technology, USA, 1:1000 dilution) or anti-osteopontin antibody (NB110-89062, Novus Biologicals, USA, 1:50 dilution) overnight, and goat anti-rabbit IgG (H + L) Fluor594 (EM35153-01, Beijing Emareio Science & Technology, China) or goat anti-mouse IgG (H + L) Fluor594 (EM35150-01, Beijing Emareio Science & Technology, China) for 1 h, while the nuclei were counterstained with DAPI. Follow-up observations were made using a 780 laser scanning confocal microscope (Zeiss, Oberkochen, Germany).

### In vivo* bone repair and evaluation*

#### Surgical procedure and scaffold implantation

A rat skull defect model was established to evaluate the repair effect of hydrogel scaffolds in vivo. SD rats (8 weeks old, Laboratory Animal Center, Sun Yat-sen University) were created with circular defects with a diameter of 5 mm on the sides of the skull after anesthesia. Then, the rats were randomly divided into 4 groups: (1) wounds without any treatment; (2) wounds treated with PLGA_PVGM/D@30nHA hydrogel; (3) wounds treated with autogenous bone; (4) wounds treated with PLGA_PVGM/D hydrogel. All the animal protocols were approved by the Institutional Animal Care and Use Committee of Sun Yat-sen University (Approval Number: SYSU-IACUC-2022-000431).

#### Micro computed tomography (micro-CT) analysis

The skulls were explanted and fixed in 4% (w/v) buffered paraformaldehyde. New bone formed in the defect area was assessed using a micro-CT system (SCANCO MEDICAL AG, Switzerland). 3D reconstructions were taken using software provided by the company (μCT evaluation Program v6.6). The ratio of bone volume to total bone volume (BV/TV), trabecular number, and bone mineral density (BMD) was quantitatively determined.

#### Histological evaluation

The slices were stained with hematoxylin and eosin (H&E), Masson’s trichrome staining, and immunohistochemical (IHC) staining which incubated with primary antibodies against Runx2 (GB11264, ServiceBio, China), Col I (GB11022-3, ServiceBio, China), and Ocn (GB11233, ServiceBio, China), respectively. Images were taken using a Leica Biosystems microscope (Aperio^®^ AT2, Germany).

#### Statistical analysis

Data are presented as mean ± standard deviation (SD). Differences between groups were analyzed by one-way analysis of variance (ANOVA). For all testing, the significance level was set at *p < 0.05, **p < 0.01, and ***p < 0.001, respectively. Statistical analysis was performed using GraphPad Prism 8 software.

## Result and disscussion

### Preparation and characterization of hydrogels

To design a hydrogel scaffold suitable for oral hard tissue, we chose two crosslinkers added to PVGM hydrogel system, respectively. The vinyl group on the benzene ring of PBA can connect the GelMA network, while the boronic acid group of its side chain and the hydroxyl group on the PVA molecule form a dynamic covalent bond. Similarly, the aldehyde group on the benzene ring of DBA can connect the GelMA network, and the catechol structure of its side chain can reinforce and stabilize the PVGM network through hydrogen bonds. Then compared the mechanical properties of these two systems. The Fourier-transform infrared spectroscopy (FTIR) spectra suggested the successful formation of PVGM, PVGM/D, and PBA crosslinked PVGM hydrogel (PVGM/P) hydrogels (Additional file 1: Fig. S1). The vinyl group on the benzene ring of PBA can be connected to the GelMA network by copolymerization, and the boronic acid group on its side chain forms a dynamic covalent bond of boronic ester bond with the hydroxyl group on the PVA molecule, which can endow the material with self-healing properties. The aldehyde group on the benzene ring of DBA could bonding the amidogen of GelMA network, and the catechol structure of its side chain can reinforce and stabilize the PVGM network through hydrogen bonds.

**Fig. 2:**
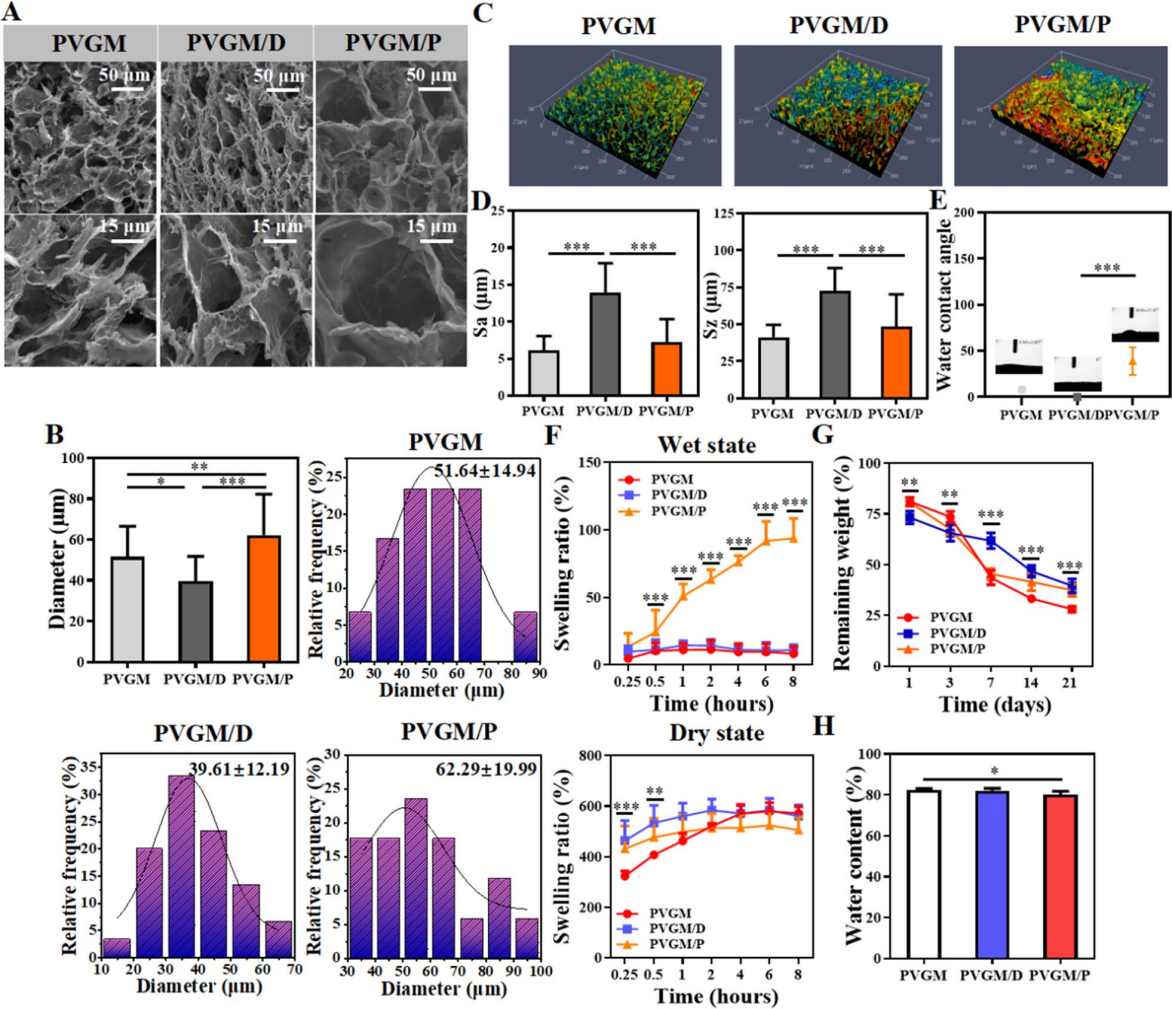
Characterizations of the different double-network hydrogel system. **A** Scanning electron microscope (SEM) images of different hydrogel scaffold; **B** the average cross-sectional pore sizes (n = 30) of different hydrogels; **C**, **D** the surface microprofile, the corresponding arithmetic average height deviation from a mean plane (Sa) and the maximum height (Sz) (n = 3) of different hydrogels; **E** water contact angles (WCAs) of the samples (n = 3); **F** the swelling ratio of the samples at wet (above) and dry (below) state; **G** the in vitro degradation rate of the hydrogels at phosphate buffered saline (PBS) solutions (n = 3); **H** water content percentages (WCPs) of the samples (n = 3) (***p < 0.001, **p < 0.01, *p < 0.05)

As shown in Fig. 2A and Additional file 1: Table S1, the results of SEM and EDS indicate that DBA and PBA were successfully incorporated into the PVGM polymer network with microporous structures. In this study, the average pore size variations (Fig. 2B, Additional file 1: Table S2) of the three groups of PVGM (51.64 ± 14.94 μm), PVGM/D (39.61 ± 12.19 μm), and PVGM/P (62.29 ± 19.99 μm) can affect the mechanical strength and surface roughness of the hydrogel, and it is expected to promote osseointegration between the materials and host bone by adjusting the pore size. The change of porous microstructure may result because the DBA, a catechol-rich polymer, was coordinated to, and distributed inside of, the PVGM. For tissue engineering, this feature can enhance the permeability of essential components and support cellular functions such as adhesion, proliferation, and differentiation [21, 26].

The quantitative analysis of Sa & Sz (Fig. 2C, D, Additional file 1: Table S3) showed that the surface of PVGM/D was significantly coarsest of these three groups. The increase in roughness may be due to the decrease in pore size (Fig. 2B). Hydrophilicity is a key parameter affecting protein adsorption, cell adhesion, and proliferation of biomaterials [27]. As shown in Fig. 2E, the three hydrogels, PVGM, PVGM/D, and PVGM/P, all displayed hydrophilicity with contact angles of less than 90°. However, the contact angle was close to zero at the moment of water dripping on the PVGM/D surface. A possible reason is that DBA has a catechol structure that forms a short-lived hydrogen bond with water on the surface of the hydrogel to enable the droplets better dispersed. The problem with in vivo implantation of PVGM hydrogel is its quick dissolution at neutral pH due to the dissociation of carboxyl and alcoholic hydroxyl groups [28]. In this study, stable crosslinking networks were formed due to the action of the crosslinker. The PVGM/D and PVGM/P hydrogels maintain the swelling stability in an aqueous solution because of the enhancement of hydrogen bonding and the limitation of the electrostatic repulsive force generated by the dissociation of GelMA carboxyl groups.

In general, with the addition of a crosslinker, the time and swelling ratio of PVGM/D and PVGM/P hydrogels to reach a swelling equilibrium in a dry state decrease by a different extent (Fig. 2F). An interpretation is that the contents of DBA and PBA introduce more hydrogen bonds and chemical crosslinks between GelMA chains, thereby restricting the diffusion of water molecules into the network. This is critical for the early stage of load bearing as an implantable tissue engineering scaffold. The swelling ratio of PVGM/P is much higher than other group at wet state (Fig. 2F). The reason is that the formation of reversible boronate ester bonds by the hydroxyl group of polyvinyl alcohol (PVA) and boronic acid of 4-vinylphenylboronic acid (PBA) reduced the drawing stress. These reversible dynamic covalent bonds act as the permanent multi-cross point under anhydrous conditions, thereby giving a high tensile strength to the hydrogel [29]. Their dynamic reversibility can be easily regulated by adjusting the pH value or ethanol concentration of the medium since borate is very sensitive to water or alcohol. Therefore, the boronic esters in water are unstable and result in the hydrolysis reaction, reducing the limitation on the hydrogel network by the boronate ester bonds. Thereby the swelling rate of the PVGM/P at wet state is higher [30].

Meanwhile, the chemical crosslinking of PBA or DBA prolongs the degradation of the double-network hydrogel system. The degradation performance of the hydrogel system in vitro was evaluated by immersion in PBS with collagense (Fig. 2G). The degradation rate of the hydrogel group added with DBA or PBA was significantly diminished at week 3 compared with the PVGM hydrogel group. The reason for the slower degradation rate of PVGM/D and PVGM/P hydrogel systems is that the double bonds between the two polymer networks and the crosslinker form a stronger and more stable network structure [25]. Furthermore, the PVGM/D group showed optimal stability.

Swelling capacity and degradation rate are two crucial factors for controlling the diffusion and release of drugs and nutrients from hydrogels and can reveal the structural characteristics and mechanical properties of hydrogels. Equilibrium water content (EWC) also represents the cross-linking density and the mechanical properties of hydrogel [31, 32] 3,4. In Fig. 2F–H, decreased swelling ratio, EWC, and degradation ratio demonstrated increased cross-linking density and better mechanical properties.

From the above results, we deduced that the hydrogel system with DBA as a crosslinker was a great choice for bone tissue filling material due to its stable polymer network structure.

**Fig. 3:**
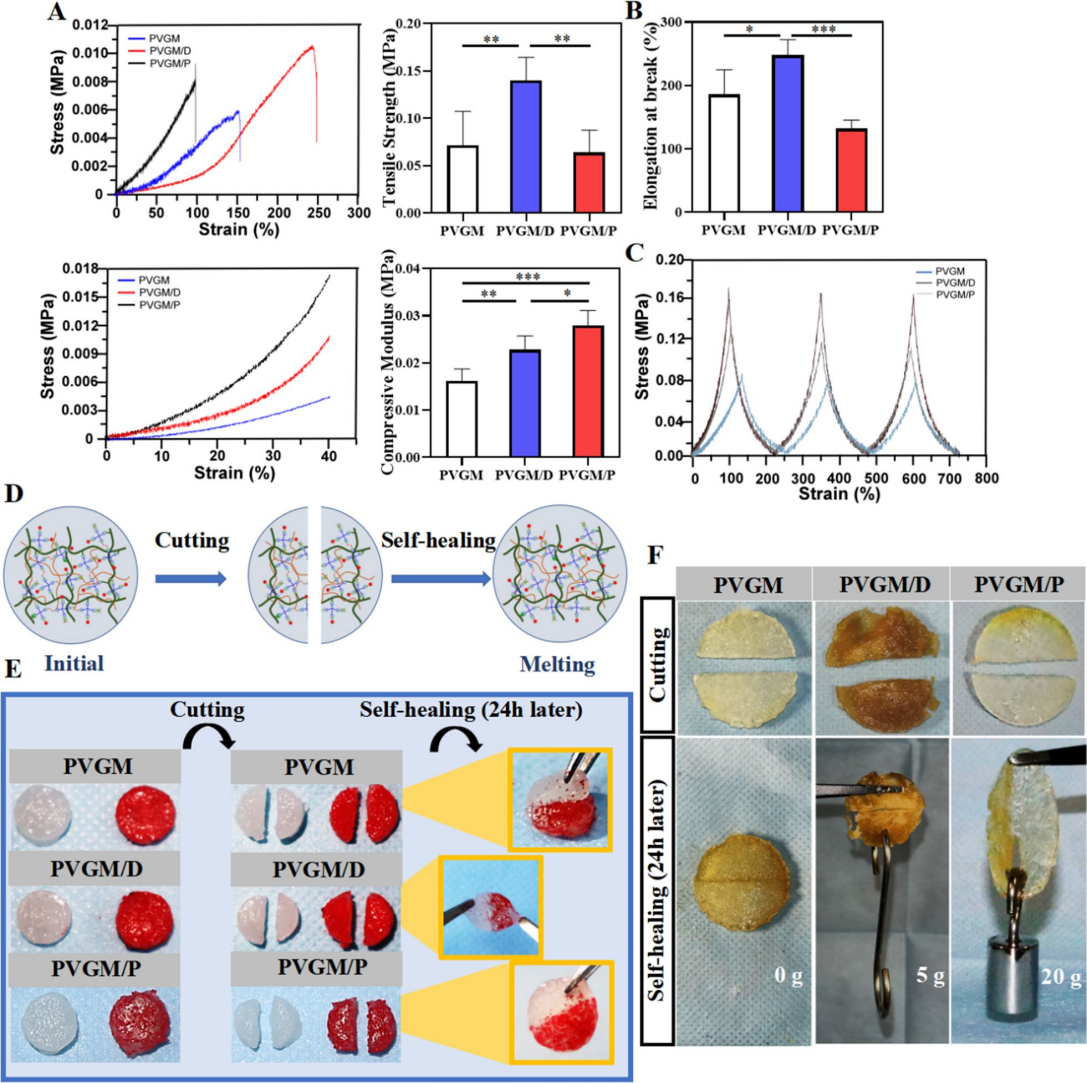
Self-healing properties and evaluation of compression and tension of hydrogels with different cross-linker. **A** The compressive stress–strain curves, tensile stress–strain curves, the compressive modulus, tensile strength at break, and **B** elongation at break of the samples calculated from mechanical testing (n = 3) of the hydrogels (***p < 0.001, **p < 0.01, *p < 0.05); **C** cyclic compressive stress–strain curves of samples; **D** Schematic illustration of the self-healing process; **E**, **F** photographs showing the self-healing behavior of two pieces of one hydrogel after 24 h of cutting.

It has been known that there is non-covalently crosslinking in the double-network PVGM hydrogel, which is composed of GelMA cross-linked network and PVA cross-linked network. Since the hydrogen bonding interaction between the two cross-linked networks which are too weak to act as a strengthening mechanism [33], the compressive modulus is still much lower than the hardness (25-40Kpa) [34] suitable for bone tissue regeneration (Fig. 3A, B). In contrast, the mechanical properties of the PVGM/D and PVGM/P hydrogels were significantly improved compared to the pristine PVGM hydrogels. For PVGM/D and PVGM/P hydrogels, the higher mechanical strength is mainly attributed to the covalent crosslinking enhancement mechanism brought by the crosslinker [35]. Since bioactive ingredients will be introduced to endow the hydrogel with biological properties and further improved the mechanical properties of the composite hydrogels by incorporating the inorganic phase, PVGM/D hydrogels with excellent tensile properties and compressive properties were selected to be used for the following experiments.

The fatigue resistance property of the hydrogels has been tested (Fig. 3C, Additional file 1: Fig. S2D). Among the three cycles, PVGM/D hydrogel has the best stress-strain curve overlap, and its elasticity and toughness are obtained from the covalent interactions due to the introduction of 3,4-dihydroxybenzaldehyde (DBA) into the PVGM double network polymer, demonstrating the formation of an energy-dissipative hybrid network.

### Self-healing ability

For the significance of self-healing ability in bone healing, the complex environment in the human body might lead to the damage of hydrogels and the failure of implantation surgery. Hence, the integrity of the hydrogel structure after implantation is crucial [36]. To overcome the vulnerability of hydrogels in vivo, hydrogels are needed to support cell function and reversible remodeling after destruction [37]. Self-healing Hydrogels can protect cells from weak interactions. And without any external stimulations, the self-healing process can occur under physiological conditions [38]. The rapid self-recovery of mechanical and structural properties ensures the spontaneous integration of the hydrogel after implantation and the repair of tiny cracks during use [39]. For hydrogels loaded with bioactive substances, the fast self-healing allows local restriction of hydrogels and incorporated compounds, resulting in higher and more sustained effective doses and reduced off-target side effects [40]. Moreover, these self-healing hydrogels by reversible covalent and non-covalent bonds, are expected to extend their application life in tissue engineering [41]. Self-healing hydrogels are typically either rapidly self-healing or mechanically stable, but not both [42]. As shown in Fig. 3D–F, when the hydrogel maintained its circular shape. These results suggested that all three hydrogels had self-healing ability in the presence or absence of a crosslinker, resulting from the dynamic noncovalent and covalent interactions involving the polymer-polymer network of the hydrogel. PVGM/D has a higher tensile strength after self-healing test compared to PVGM or PVGM/P, which is more conducive to maintaining the integrity of the scaffold material and the stability of the osteogenic space.

### Properties of hydrogels incorporating nHA

**Fig. 4:**
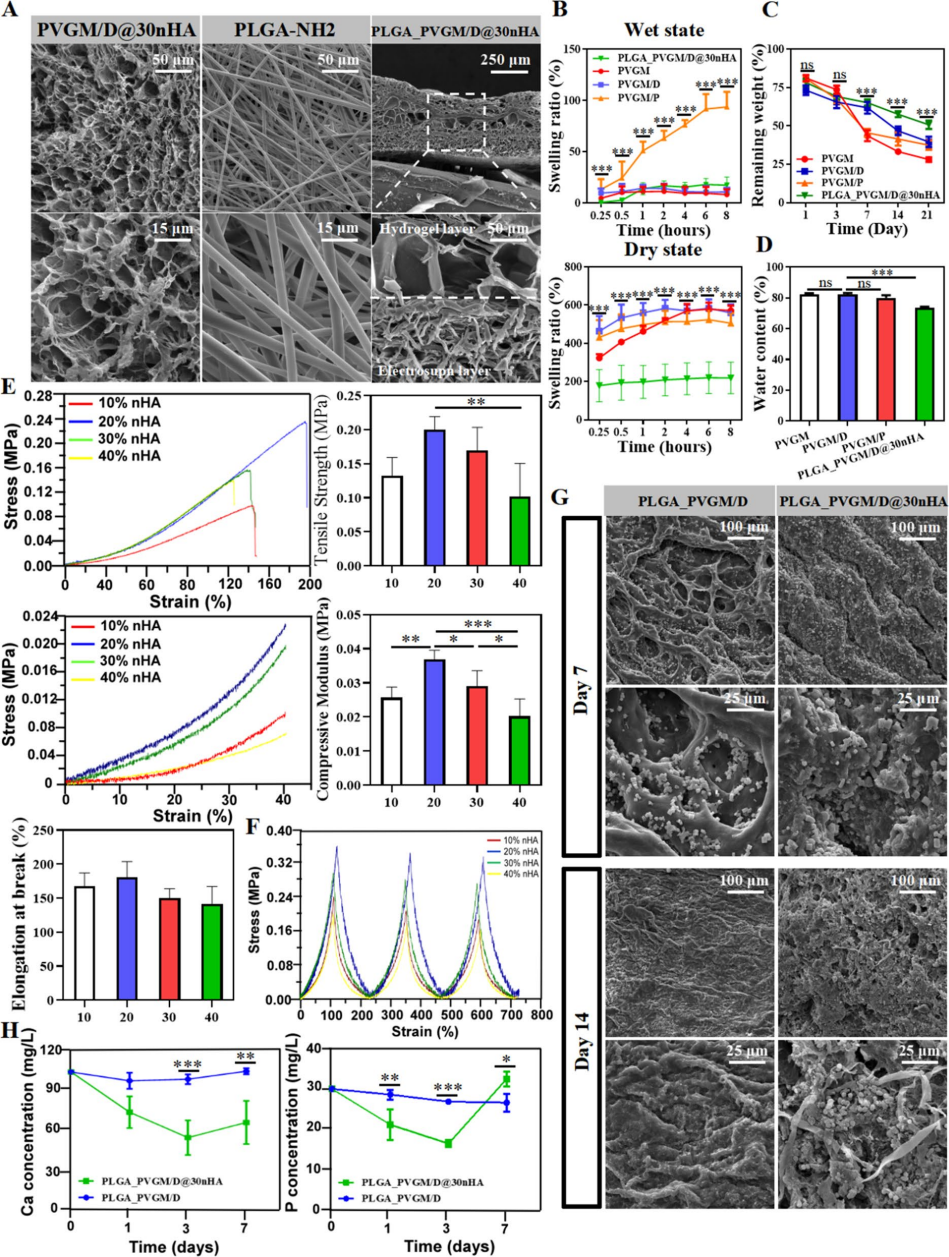
Evaluation of compression and tension of hydrogels with different content of nHA and mineralization behaviors of PVGM/D and PLGA_PVGM/D@30nHA. **A** The cross-sectional SEM of electrospun PLGA fiber and PLGA_PVGM/D@30nHA; **B** the swelling ratio of the samples at wet (above) and dry (below) state; **C** the in vitro degradation rate of the hydrogels at phosphate buffered saline (PBS) solutions (n = 3); **D** water content percentages (WCPs) of the samples (n = 3); **E** tensile stress − strain curves, tensile strength at break, compressive stress–strain curves and compressive modulus of the samples calculated from mechanical testing (n = 3); **F** cyclic compressive stress–strain curves of samples; **G** the SEM images of crystallization on different surfaces after immersing in simulated body fluid (SBF) for 7 days and 14 days; **H** the Ca and P ions release of different hydrogels after soaking in SBF for 1, 3, and 7 days. ***p < 0.001, **p < 0.01, *p < 0.05

Due to the high water content, hydrogels are semi-permeable to small molecules and ions, and even to some proteins [43, 44]. Semi-permeability enables the mineralization of HA nanocrystals in hydrogels [45]. Thus, combining the features of the semi-permeability of the hydrogels, the high osteoconductivity of HA, and the dynamic nature of the bone tissues, HA-containing hydrogels can spontaneously induce the formation of a gel/hybrid layer at the gel-bone interface in vivo to promote bone regeneration. Thereby, we fabricated PVGM/D@nHA composites by adding nHA to PVGM/D hydrogels. Taking PVGM/D@30nHA for example, the FTIR spectrum showed that nHA was successfully doped into the hydrogel system (Additional file 1: Fig. S1). To optimize the composition of PVGM/D@nHA hydrogels for bone regeneration, Hydrogels were fabricated with varying nHA content. Figure 4A shows the morphology of a representative PVGM/D@30nHA hydrogel scaffold, in which the ordered and uniform macropores are interconnected, and the pore diameter decreases after adding nHA.

To impart soft tissue barrier properties to the PVGM/D@nHA, a PLGA fiber layer was electrospun as a barrier to isolate bone defects from soft tissue. The amino group modifying PLGA bonding to the hydrogel by aldol-amine condensation with the aldehyde group of DBA. As shown in Fig. 4A and Additional file 1: Table S2, the cross-sectional images show that the two layers are tightly integrated without gaps. The hydrogel produced by the chemical cross-linking method is loose and porous as well as the electrospun membrane. The surface microprofile, and the quantitative analysis of Sa & Sz Additional file 1: Fig. S2A, Table S3) demonstrated that the introduction of nHA does not affect roughness, and PVGM/D@30nHA maintains high protein adsorption and cell adhesion ability. Furthermore, it was observed that the pore size of the hydrogel close to the interface became larger (75.10 ± 24.45 μm) (Additional file 1: Fig. S2B). As shown in Additional file 1: Fig. S2C, PVGM/D@30nHA was hydrophilic, which can not support the water droplets on its surface. The PLGA electrospun fiber layer of PLGA_PVGM/D@nHA was hydrophobic with a contact angle of 131.1 ± 7.12°. The better the hydrophilicity, the better the properties of protein adsorption, cell adhesion, and proliferation of biomaterials [46]. Thus, the hydrophobicity of the PLGA membrane was beneficial to realizing soft tissue barrier function.

With the introduction of the nHA, the swelling, degradation, and EWC ratios of the hydrogel, and the swelling equilibrium time further reduced significantly (Fig. 4B–D). Because the nHA formed coordination bonds with the hydroxyl groups carried by the PVGM/D hydrogels to restrict the diffusion of water molecules into the network To ensure that the tissue structure can be incorporated into the surrounding host tissue, it requires that biopolymer scaffolds degrade at a rate similar to the growth rate of new tissue as a mismatch in these rates can lead to premature failure of the tissue development [47]. However, the optimal degradation rate is unclear because bone regeneration is affected by multiple factors. The conditions of different patients, the surgical methods, and the size of the defect area all determine the osteogenesis speed. Here we take PLGA_PVGM/D@30nHA hydrogel as an example, which has good biodegradability in PBS with suitable mass loss. The mass loss in PBS indicated its excellent resistance to hydrolysis (Fig. 4C). The reason for the slower degradation rate of the nanocomposite hydrogel system is the formation of a stronger and more stable network structure by the covalent network between the GelMA and the PVA. The coordination bonds between the calcium ions carried by nHA and the hydroxyl groups carried in PVGM/D formed a higher crosslink density to effectively decrease the degradation rate and improve the resistance to degradation of the nano-double network hydrogel.

It was observed that the mechanical strength increases at first and then decreases with increasing nHA content (Fig. 4E). Although the introduction of nHA does not affect chemical crosslinking, it may influence the formation of hydrogen bonds as well as coordination bonds in PVGM/D. The incorporation of nHA significantly enhanced the mechanical properties of PVGM/D, since the incorporation of HAP poly-crystals as “sacrificial bonds” into a double network gel consisting of two identical neutral polymers could significantly increase the toughness of the gel [22].

However, the mechanical strength of PVGM/D@nHA did not change significantly at lower or higher concentrations of PVGM/D@nHA (nHA content of 10% or 40%). A possible explanation for the observed lack of concentration effect on the compressive modulus is that the increase in nHA concentration might induce catechol-Ca coordination while simultaneously reducing the availability of the catechol groups required for crosslinking between polymer chains [48]. Increasing the nHA concentration, the tensile strength and compressive modulus decreased. However, the mechanical properties of PVGM/D@20nHA and PVGM/D@30nHA both met the requirement for bone tissue regeneration and osteogenic differentiation of BMSCs [34]. Therefore, in the subsequent experiments, PVGM/D hydrogels doped with 10%, 20%, and 30% nHA were chosen to explore their biological functions.

The PVGM/D@nHA hydrogels were resilient and tough because the nHA poly-crystals introduced noncovalent interactions into the covalently cross-linked PVGM/D network, and formed an energy-dissipative hybrid network. No fractures were observed in the process of measurement of the 3-cycle compression of four hydrogels (Fig. 4E, Additional file 1: Fig. S2), and the PVGM/D@nHA hydrogel gradually recovered to its original shape after relaxation, proving that formation of homogeneous catechol-Ca coordination. Meanwhile, the compressible and reversible properties of the prepared samples are demonstrated by the narrow gap formed by the first and last compression/relaxation curve. Figure 4F and Additional file 1: Fig. S2D show the fatigue resistance of each group: 20% > 30% > 10% > 40%, which is consistent with compressive strength, respectively. In clinical practice, the hydrogel should easily be cut and placed according to the shape of the defect, which requires the hydrogel to have good plasticity. After oral implantation, the compressive strength, and anti-fatigue properties of hydrogels are crucial for maintaining the osteogenic space, considering the physiological activities that happen in the oral cavity.

### Ions release and apatite-forming ability

The ideal material system should be mineralized to induce the formation of crystals and bind to the host bone [49]. Apparent sediment was observed along the surface of the PLGA_PVGM/D@30nHA hydrogel, and its amount increased with time, suggesting that it has a strong apatite-forming ability (Fig. 4G). The ion release (Fig. 4H) results also verified that the apatite formed by the changing trend of calcium and phosphorus in the first 7 days. After a sharp decrease, the calcium concentrations in the PLGA_PVGM/D@30nHA solution increased while remaining relatively stable in the PLGA_PVGM/D group. It was due to the rate of apatite-formation by calcium deposition in the SBF solution of nHA in the early stage being higher than that of calcium release and calcium dissolution, and then the partial dissolving of apatite happened [50]. For the same reason, the content of phosphorus ions changed as the same trend and almost without changes in PLGA_PVGM/D group. The mineralization test showed that the hydrogel had good bioactivity due to the addition of nHA. The dispersed nHA also act as nucleation sites for the promotion of hydroxyapatite (HA) precipitation as well as cell adhesion sites that enable integration with surrounding bone tissue [51, 52]. The PLGA_PVGM/D@30nHA group showed optimal stability and sustained release over 7d (Fig. 4G, H), and the concentrations of released calcium and phosphorus ions could be easily mediated by adjusting the coordination between PVGM/D and nHA. From the above results, we deduced that the nHA-incorporated hydrogel system was a great choice for long-term bone tissue engineering (BTE) due to its stable polymer network structure and efficient delivery and tailored release of Ca^2+^ and PO_4_^3−^.

### Barrier function

**Fig. 5:**
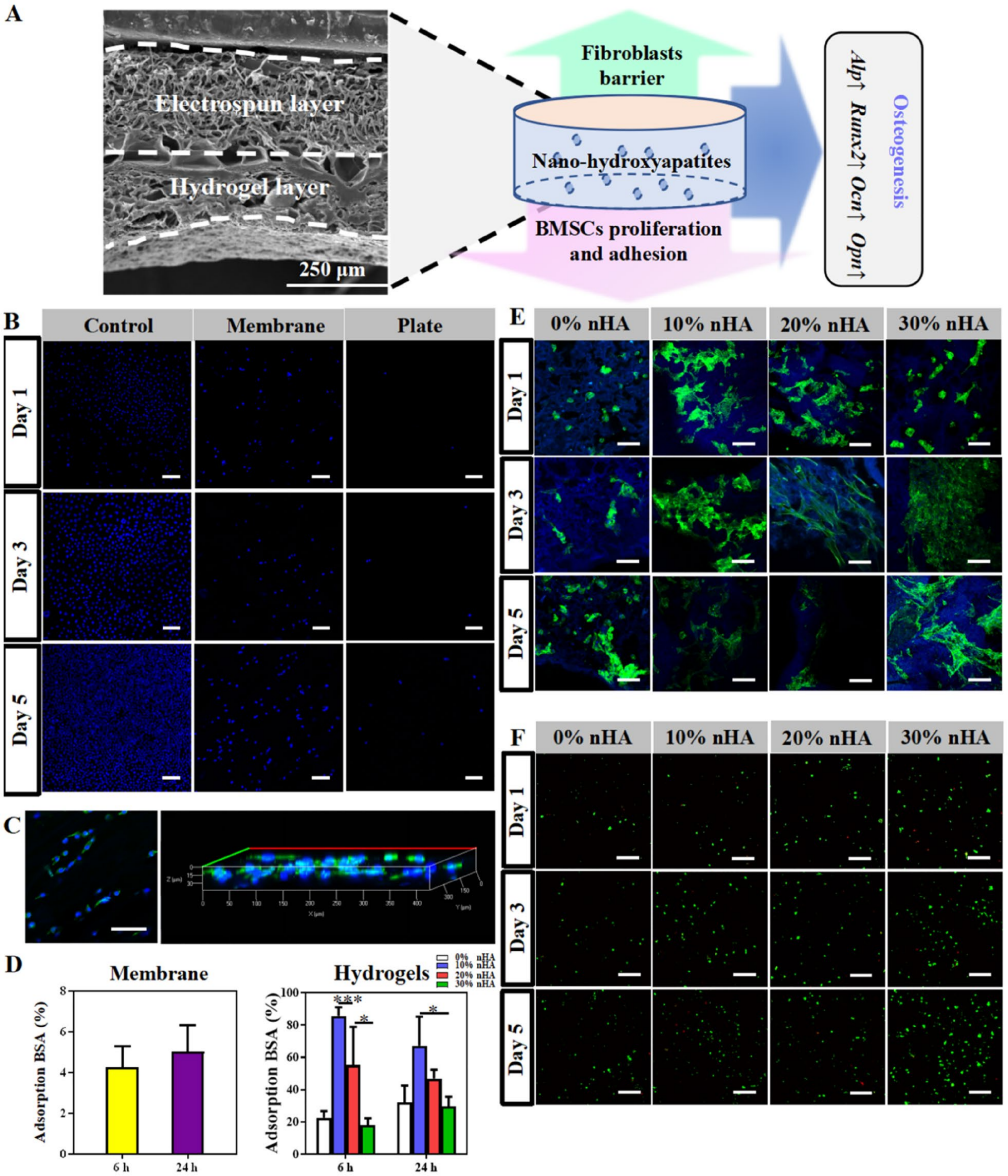
BMSCs adhesion on the hydrogels and cells penetration analysis on electrospun membrane (n = 3). **A** Schematic illustration of the cell barrier function and cell behaviors in the stage of bone regeneration; **B** the confocal scanning of the control chambers, the 24-well plate, and the electrospun membrane after 1, 3 and 5 days, the scale bar is 200 μm; **C** confocal image and 3D reconstruction of the L929s cultured on the membrane surface after 5 days; **D** the BSA adsorption at 6 and 24 h of hydrogels and electrospun membrane; **E** the BMSCs morphology after cultured for 1, 3 and 5 days. The scale bar is 100 μm; **F** The live/dead staining of BMSCs cultured for 1, 3 and 5 days. The scale bar is 200 μm. BMSCs osteogenesis analysis in *vitro*. **A** The ALP staining and **B** activityon the 4th and the 7th day (n = 3); **C** the Alizarin red S staining was carried out on the 14th day; **D** the relative mRNA expressions of Runx2 and Alp in BMSCs cultured on different hydrogels for 4 and 7 days, while Ocn and Opn for 7 and 14 days (n = 3); **E** the Runx2 immunofluorescent staining of BMSCs cultured on different hydrogels for 4 days and 7 days and the Opn for 7 days and 14 days (n = 3). The scale bar is 200 μm. ***p < 0.001, **p < 0.01, *p < 0.05

As the schematic illustration shown, the original intention of the hierarchical scaffold was to obstruct the entry of fibroblasts and epithelial cells into the osteogenic space using a barrier membrane (Fig. 5A). In our design, the surface as a barrier consists only of PLGA carrying amino groups. Hence, we only investigated the barrier function of the PLGA electrospun membrane. The Control group was the bottom surface of the 12-well plate without membrane, with only L929s added into the well. The membrane group was the surface of the PLGA electrospun membrane placed on the bottom of the well with L929s seeded on the membrane surface. The Plate group was the bottom surface of the well after taking away the membrane of the Membrane group. We found a small number of L929s on the surface of the PLGA electrospinning membrane compared with the Control group, indicating that the membrane was not conducive to cell adhesion. While in the Plate group, we observed several L929s on the bottom surface of the well, indicating that the electrospun membrane prevented most of the penetration of L929s, and only a few L929s penetrated through the membrane surface to the bottom of the well. As the result showed (Fig. 5B), in the group with the PLGA electrospun membrane fixed in the transwell chambers, only a few cells were visible on the surface of the membrane and not on the bottom of the plates, which prevented cell infiltration. Through confocal scans and 3D reconstructed images of membranes (Fig. 5C), we also observed that L929s remained on the electrospun membrane surface instead of migrating extensively into the membrane. In conclusion, the chemically crosslinked PLGA electrospun membrane bound on the hydrogel surface successfully created a barrier between soft and hard tissues, effectively resisting the infiltration of connective tissue, and establishing a suitable microenvironment for osteogenesis-related cells to form new bone.

### Biofunctions

The adsorption of BSA, the proliferation, and the adhesion of BMSCs on rough surfaces were investigated to evaluate the biocompatibility of composite hydrogels. Besides the PVGM/D@10nHA group, the adsorption rate of BSA showed no significant difference among the other three groups (Fig. 5D). The images of the cellular adhesion morphology on the 1st, 3rd, and 5th days showed that the cells stretched on the surfaces and displayed a spindle shape, indicating proper cellular attachment and viability in different content of nHA microenvironments. Meanwhile, the area of BMSCs spread and stretched on the hydrogel surface in the PLGA_PVGM/D@20nHA group was larger than in the PLGA_PVGM/D@30nHA group, indicating that the reduction in cell spreading area in the PLGA_PVGM/D@30nHA group may be associated with cell differentiation [53] (Fig. 5E, Additional file 1: Fig. S3). Moreover, Live/Dead staining fluorescent (Fig. 5F, Additional file 1: Fig. S4A) images and the CCK-8 results (Additional file 1: Fig. S4B) showed that BMSCs in the PLGA_PVGM/D@30nHA group proliferated with time, and the PLGA_PVGM/D@30nHA significantly promoted cell growth on the 5th day (Fig. 5F, Additional file 1: Fig. S4). This result is similar to other reported studies [54, 55]. One possible reason could be the ions released from nHA, such as calcium and phosphorus, facilitating the cell activity. However, the other three groups showed a slight inhibitory effect on cell proliferation with time. It is probably due to the acidic microenvironment created by hydrogel degradation [56], while the alkaline microenvironment from ion exchange in nHA degradation can not be completely neutralized. Considering the biological effects, the PLGA_PVGM/D@nHA hydrogels with good biocompatibility are expected to be a scaffold for cell growth and osteogenesis.

### In vitro* osteochondral repair efficacy*

**Fig. 6:**
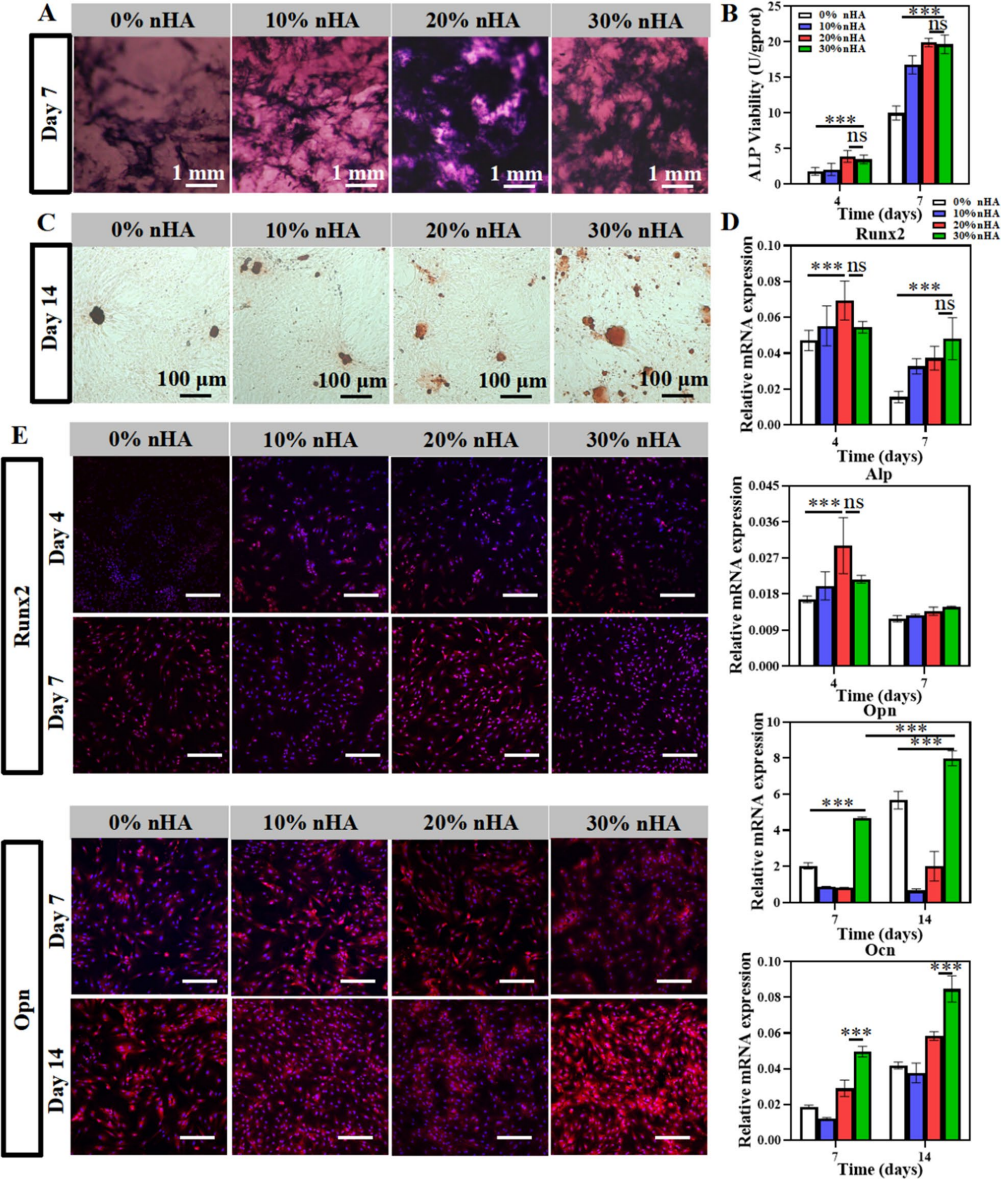
BMSCs osteogenesis analysis in *vitro*. **A** The ALP staining and **B** activityon the 4th and the 7th day (n = 3); **C** the Alizarin red S staining was carried out on the 14th day; **D** the relative mRNA expressions of Runx2 and Alp in BMSCs cultured on different hydrogels for 4 and 7 days, while Ocn and Opn for 7 and 14 days (n = 3); **E** the Runx2 immunofluorescent staining of BMSCs cultured on different hydrogels for 4 days and 7 days and the Opn for 7 days and 14 days (n = 3). The scale bar is 200 μm. ***p < 0.001, **p < 0.01, *p < 0.05

We next tested alkaline phosphatase (ALP) activity, ALP expression; alkaline phosphatase (Alp), Runx-related transcription factor 2 (Runx2), osteopontin (Opn), and osteocalcin (Ocn) gene expression; and the immunofluorescence staining of Runx2 and Opn to observe the hydrogel-derived impact on BMSC osteogenic differentiation. The ALP staining result was more positively expressed in the PLGA_PVGM/D@nHA group with 20% and 30% contents at each observation point, but there were no significant differences between them (Fig. 6A). A similar trend was observed in the ALP activity (Fig. 6B). ARS staining (Fig. 6C) result showed that the highest level of staining was observed in the PLGA_PVGM/D@30nHA group, indicating osteogenic capacity enhancement at the late stage. On the 4th day, the qPCR expression levels of Runx2 and Alp were significantly enhanced in PLGA_PVGM/D@20nHA but were similar with PLGA_PVGM/D@30nHA. Additionally, on the 7th and 14th days, PLGA_PVGM/D@30nHA exhibited the most assertive promotion in the expression of Opn and Ocn (Fig. 6D). These differential trends could be ascribed to the different stimulatory effects of hydrogels with different stiffnesses on BMSCs. Many factors affect BMSC osteogenic differentiation, including extracellular matrix stiffness [57], drugs [58, 59], bioactive substances [54, 60], etc. According to our results and relevant studies, we suggest that BMSCs are more likely to be stimulated by an extracellular matrix with higher stiffness in the early stages of osteogenic differentiation [61]. In the late stage of osteogenic differentiation, the content of bioactive components determines the expression level of osteoblast protein in BMSCs. Relevant studies have confirmed that 30% nHA is more conducive to osteogenic differentiation [54, 62].

Within the suitable stiffness for bone regeneration, BMSCs could be more susceptible to early stimulation on the surface of hydrogels with higher stiffness [61]. Moreover, the confocal images showed that the fluorescence intensity of Runx2 (7th day) and OPN (14th day) increased with the addition of nHA in the PLGA_PVGM/D@30nHA group (Fig. 6E). The above results demonstrate that the addition of nHA enhances the osteogenic ability of the hydrogel, which is similar to other studies [55, 63, 64]. It is suggested that the sustained release of nHA loaded by PLGA_PVGM/D@30nHA hydrogel scaffold may be beneficial to accelerating the osteogenic differentiation of BMSCs. Morever, the nHA concentration is critical, although it has an excellent ability to induce bone regeneration and osseointegration [65].

### Bone repair efficacy

**Fig. 7:**
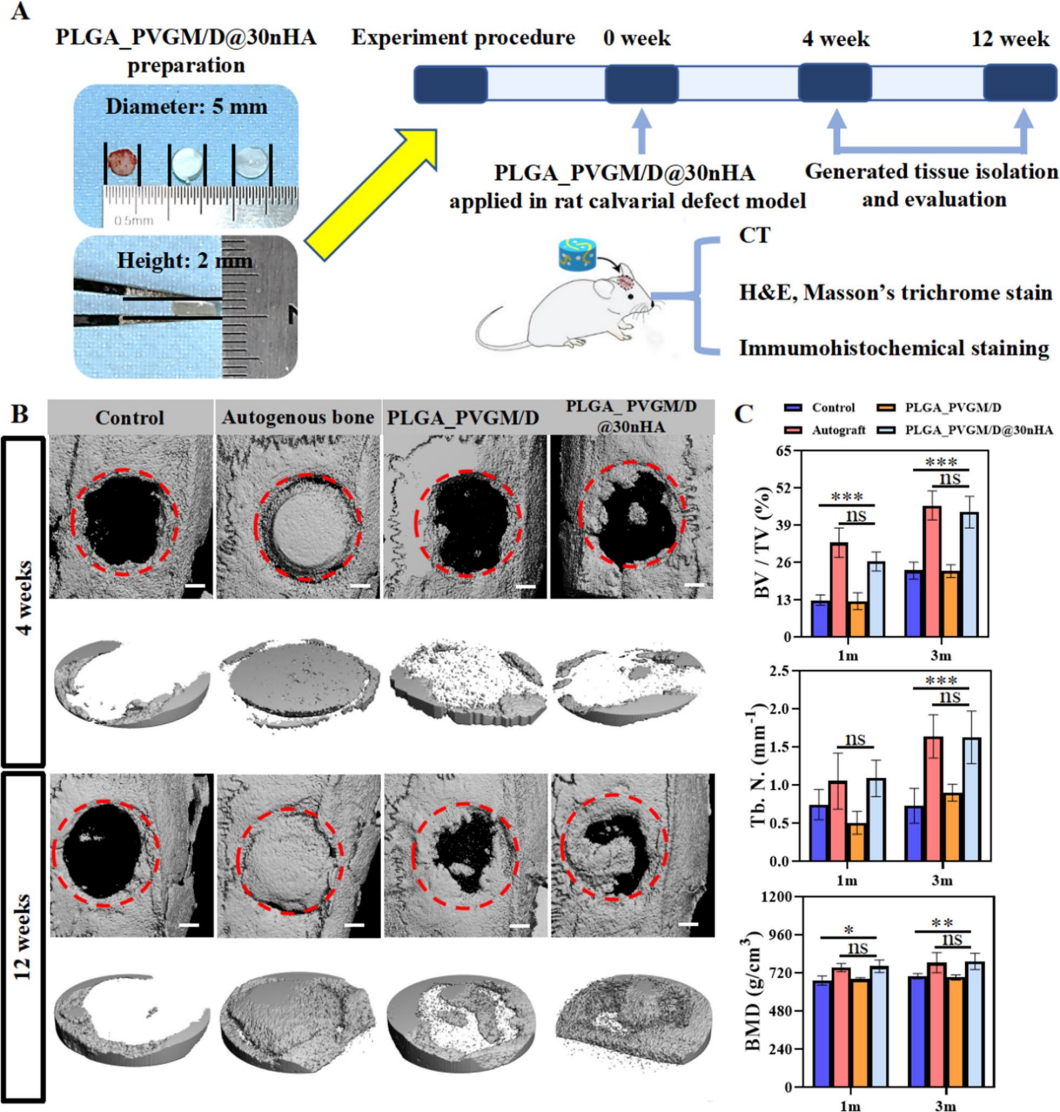
PLGA_PVGM/D@30nHA hydrogels as bone substitutes for bone regeneration in calvarial defect. **A** Schematic of the experimental procedure using PLGA_PVGM/D@30nHA hydrogels for rat calvarial defect treatment; **B** microcomputed tomography images and new bone of calvarial defects treated with different hydrogels, autogenous bone or empty at 4 weeks and 12 weeks after surgery. The scale bar = 1 mm; **C** The μCT quantification of bone regeneration in calvarial defects. Micromorphometric bone parameters of the calvarial defects after treatment including relative bone volume (bone volume of regenerated tissue/total bone volume of wound site × 100%, BV/TV%), the trabecular number (Tb.N.) and bone mineral density (BMD) in the VOI (n = 5) (***p < 0.001, **p < 0.01, *p < 0.05)

Through mechanical tests, we found that the strength of the hydrogel group with more than 30%nHA concentration decreased. In biological experiments, we found that the 30%nHA hydrogel group was the most beneficial to the osteogenic differentiation of BMSCs. Therefore, we focus on the PLGA_PVGM/D@30nHA hydrogel in ions release. As other nHA concentration groups were set up as control groups to highlight the advantages of the experimental group, we did not conduct many studies on them. In *vivo* studies, we selected the PLGA_PVGM/D@30nHA hydrogel as the experimental group. The surgery procedure was showed in Fig. 7A. Micro-computed tomography (Micro-CT) images and 3D reconstruction were taken after surgery to evaluate the new bone formation (Fig. 7B). At 12 weeks postimplantation, newly formed bone was particularly pronounced in the PLGA_PVGM/D@30nHA scaffold group, where the entire defect area was filled with new bone. The bone repair effect of the autogenous bone group was also improved, but not significantly different from that of the PLGA_PVGM/D@30nHA scaffold group. In contrast, the defects in the untreated group had little new bone formation along the margin of implantation. Quantitative analysis data of newly formed bone within the defect region further confirm the micro-CT findings (Fig. 7C). At 4 weeks, the bone mineral density of the PLGA_PVGM/D@30nHA scaffold group was significantly higher than that of the autogenous bone group, indicating that there was bone resorption in the autogenous bone group. The values of the ratio of bone volume to tissue volume (BV/TV), bone mineral density (BMD), and trabecular number (Tb. N) of the PLGA_PVGM/D@30nHA scaffold group are significantly higher than in the untreated group and the PLGA_PVGM/D scaffold group at different point of time. These results demonstrate that the PLGA_PVGM/D@30nHA scaffold can play an important role in osteoconductivity and mechanical support, and can further accelerate bone regeneration after ions release.

**Fig. 8:**
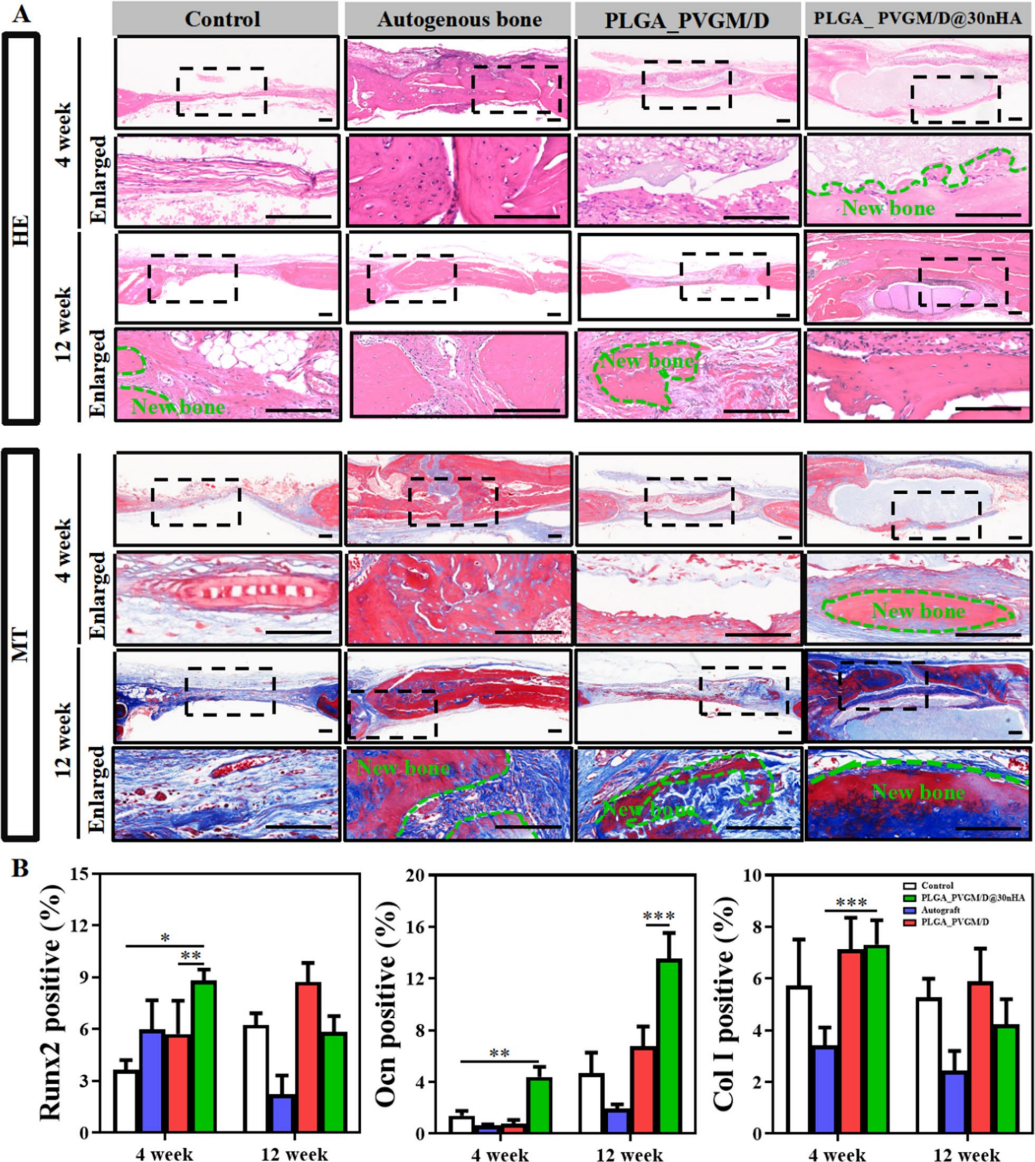
Histological evaluation of bone regeneration and osteoblasts at 4 and 12 weeks in different groups. **A** Hematoxylin-eosin (H&E) staining and masson’s trichrome (MT) staining of newly generated bone tissue in the PLGA_PVGM/D@30% nHA group, compared to the PLGA_PVGM/D group, the autogenous bone group and the untreated control (blank) group at 4 and 12 weeks. The black dotted boxes in the upper panels were enlarged in the lower panels. The area marked by the dotted green line was new bone tissue; **B** the immunohistological staining quantification of Collagen I, Ocn or Runx 2 positive stained area percentage. The scale bar is 200 μm. ***p < 0.001, **p < 0.01, *p < 0.05

**Fig. 9:**
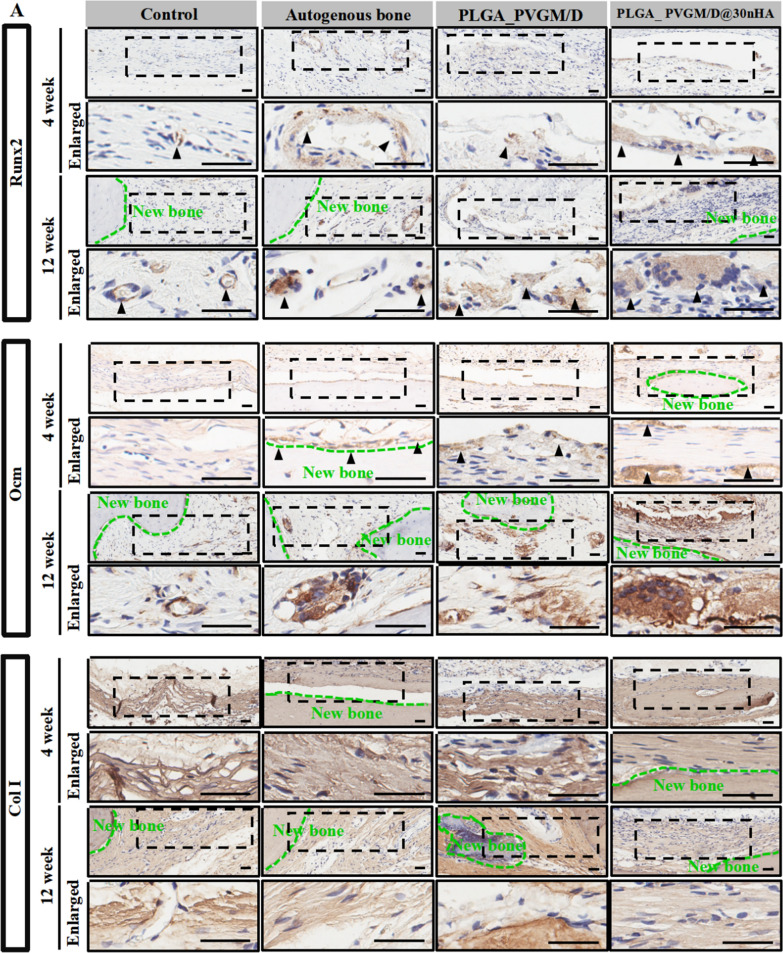
Histological evaluation of bone regeneration and osteoblasts at 4 and 12 weeks in different groups. **A** The immunohistological staining of Runx2, Ocn, and Col I of newly generated bone tissue in the PLGA_30% nHA/DNH group, compared to the PLGA_PVGM/D group, the autogenous bone group and the untreated control (blank) at 4 and 12 weeks. The black dotted boxes in the upper panels were enlarged in the lower panels. The area marked by the dotted green line was new bone tissue, and the black triangle indicates positively stained cells (n = 5). The scale bar is 200 μm

Histological staining analysis further confirms that compared with the PLGA_PVGM/D scaffold and the untreated control, the PLGA_PVGM/D@30nHA scaffold can simultaneously prevent the infiltration of most of the fibrous tissue and promote bone repair (Fig. 8A). Hematoxylin-eosin (H&E) and Masson's staining show that new bone tissue in the PLGA_PVGM/D@30nHA scaffold group could be observed at the edges and middle of the defect area at 4 weeks postimplantation. At 12 weeks postimplantation, a layer of new bone tissue was seen in the bone defect region treated with PLGA_PVGM/D@30nHA group, with a thickness similar to that of the adjacent bone. The regenerated bone tissue is well integrated with the host tissue. In contrast, the defects treated with the PLGA_PVGM/D scaffold show less effective bone repair with poor quality even at 12 weeks; the defects treated with the autogenous bone show poor integration with the adjacent bone; while the untreated defects show cavities and fibrous tissue in them, with the collapse of the adjacent area (Fig. 8A). The results of IHC staining and semi-quantitative analysis were consistent with those of Micro-CT, H&E and Masson’s staining. (Fig. 9A). Protein Ocn in the bone defect region of the PLGA_PVGM/D@30nHA scaffold group was positively stained, which was significantly stronger than that of the other three groups. Collectively, PLGA_PVGM/D@30nHA scaffold can effectively accelerate bone tissue regeneration.

## Conclusion

In this study, a novel bi-layered self-healing crosslinked double-network hydrogels were successfully prepared by copolymerization of PVA and GelMA through 3,4-dihydroxybenzaldehyde (DBA). The multiple-dynamic-bond formed by the catechol structure of DBA could considerably strengthen the mechanical strength and self-healing properties of hydrogels without undermining its biocompatibility. The polymerization was initiated to form the hydrogel layer combined with electrospun layer by chemical crosslinking. The mechanically strengthened composite scaffold can provide mechanical support in the early stage of bone repair while preventing soft tissue infiltration. Incorporating nHA could promote the proliferation, ALP activities and differentiation, and osteogenic protein expression of BMSCs. The resultant hierarchical hydrogel scaffold showed superior performance for accelerating bone repair in rat calvarial defects. We believe that this bi-layered double-network hydrogel can be extended to the treatment of other load-bearing tissue defects.

## Supplementary Information

**Additional file 1: Fig S1.** FTIR spectra of PVGM, PVGM/D, PVGM/P, PVGM/D@30nHA and PLGA_PVGM/D@30nHA. **Fig S2.** Cyclic compressive stress-strain curves of samples. **Fig S3.** The BMSCs morphology after cultured on different hydrogels for 1, 3 and 5 days. The scale bar is 100 μm. **Fig S4.** The cck8, live/dead staining and 3D reconstruction of BMSCs cultured for 1, 3 and 5 days. The scale bar is 200 μm. **Table S1.** Contents of surface elements. **Table S2.** The pore sizes of the hydrogel scaffold and the fiber sizes of hydrophobic layer. **Table S3.** The visualizations of the surface roughness. **Table S4.** Primer sequences used for PCR measurements.

## Data Availability

This published article includes all data generated and analyzed during this research.
