# Supplementary material for: Self-healing hybrid hydrogels with sustained bioactive components release for guided bone regeneration
Source: J Nanobiotechnology. 2023 Feb 22;21:62. doi: 10.1186/s12951-023-01811-8 (PMC9948527; doi:10.1186/s12951-023-01811-8)
Supplement: Supplementary file 1 — Additional file 1: Fig S1. FTIR spectra of PVGM, PVGM/D, PVGM/P, PVGM/D@30nHA and PLGA_PVGM/D@30nHA. Fig S2. Cyclic compressive stress-strain curves of samples. Fig S3. The BMSCs morphology after cultured on different hydrogels for 1, 3 and 5 days. The scale bar is 100 μm. Fig S4. The cck8, live/dead staining and 3D reconstruction of BMSCs cultured for 1, 3 and 5 days. The scale bar is 200 μm. Table S1. Contents of surface elements. Table S2. The pore sizes of the hydrogel scaffold and the fiber sizes of hydrophobic layer. Table S3. The visualizations of the surface roughness. Table S4. Primer sequences used for PCR measurements. [file 12951_2023_1811_MOESM1_ESM.docx]

**Additional file 1**

**Self-healing hybrid hydrogels with sustained bioactive components release for guided bone regeneration**

Jiaxin Li^1,2,3†^, Weichang Li^1,2†^, Mengjie Kong^1,2,3^, Zongtai Li^1,2,3^, Tao Yang^1,2^, Qinmei Wang^4^* and Wei Teng^1,2^*

1 Hospital of Stomatology, Sun Yat-sen University, Guangzhou 510055, P. R. China

2 Guangdong Provincial Key Laboratory of Stomatology, Guangzhou 510055, P. R. China

3 Guanghua School of Stomatology, Sun Yat-sen University, Guangzhou 510055, P. R. China

4 Laboratory of Biomaterials, Key Laboratory on Assisted Circulation, Ministry of Health, Cardiovascular Division, First Affiliated Hospital, Sun Yat-sen University, Guangzhou, Guangdong, PR China

†These authors contributed equally to this work.

*Corresponding author:

(1) Wei Teng, Hospital of Stomatology, Sun Yat-sen University,Guangdong Provincial Key Laboratory of Stomatology, Guangzhou 510055, P. R. China.

Phone Number: 86-020-8374-2770

Email: [tengwei@mail.sysu.edu.cn](mailto:tengwei@mail.sysu.edu.cn)

1. Qinmei Wang, Laboratory of Biomaterials, Key Laboratory on Assisted Circulation, Ministry of Health, Cardiovascular Division, First Affiliated Hospital, Sun Yat-sen University, Guangzhou, Guangdong, PR China.

Phone Number: 86-020-8374-2770

Email: [wangqinm@mail.sysu.edu.cn](mailto:wangqinm@mail.sysu.edu.cn)

**Additional Figures and Tables**

**Fig. S1** FTIR spectra of PVGM, PVGM/D, PVGM/P, PVGM/D@30nHA and PLGA_PVGM/D@30nHA.

**Fig. S1** shows FTIR spectra of the hydrogels and BG. The strong absorption peak at 3314 cm^−1^ of the PVGM hydrogel group indicates the stretching vibration of N-H bands (amide A), and the characteristic peaks at 1653 cm^−1^ are assigned to the stretching vibration of C=O (amide I), FTIR spectra of PVGM hydrogel showed that the hydrogel was rich in amino and amide groups, which confirmed the high hydrophilicity of the hydrogel simultaneously.

In the PVGM/D group, the formation of hydrogen bonds between the aldehyde group and the alcohol hydroxyl group averages the electron cloud density, thereby reducing the overall stretching vibration peaks. Pure nHA shows peaks for phosphate groups at 1104, 1034, 962, 603, and 567 cm^-1^, and peaks for hydroxyl groups at 3570 cm^-1^ and 634 cm^-1^. In the spectrum of PVGM/D@30nHA, except for the disappearance of the peaks at 634 cm^−1^ and 567 cm^−1^, the remaining peaks were shifted to 1428, 1090, and 852 cm^−1^ (phosphate group), respectively. The hydroxyl peaks overlap with the amine peak which shifted to 3314 cm^−1^. The absorption bands of PVGM/P at 1411, 1300 and 1088 cm^−1^ are assignable to B-O-H deformation, B-O stretching, and B-C stretching vibrations, respectively. It contains an absorption band due to the B-O-H in the boronic acid esters at 913 cm^−1^. In the PLGA_PVGM/D@30nHA group, the characteristic peaks of PLGA at 1750 and 1173 cm^−1^ are assigned to the stretching vibration of C=O and C-O, respectively. Due to the aldol-amine condensation reaction between the amino group carried by PLGA and the aldehyde group in benzaldehyde, the peak appearing at 1545 cm−1 is ascribed to the stretching vibration of C-N-H in the amide bond.


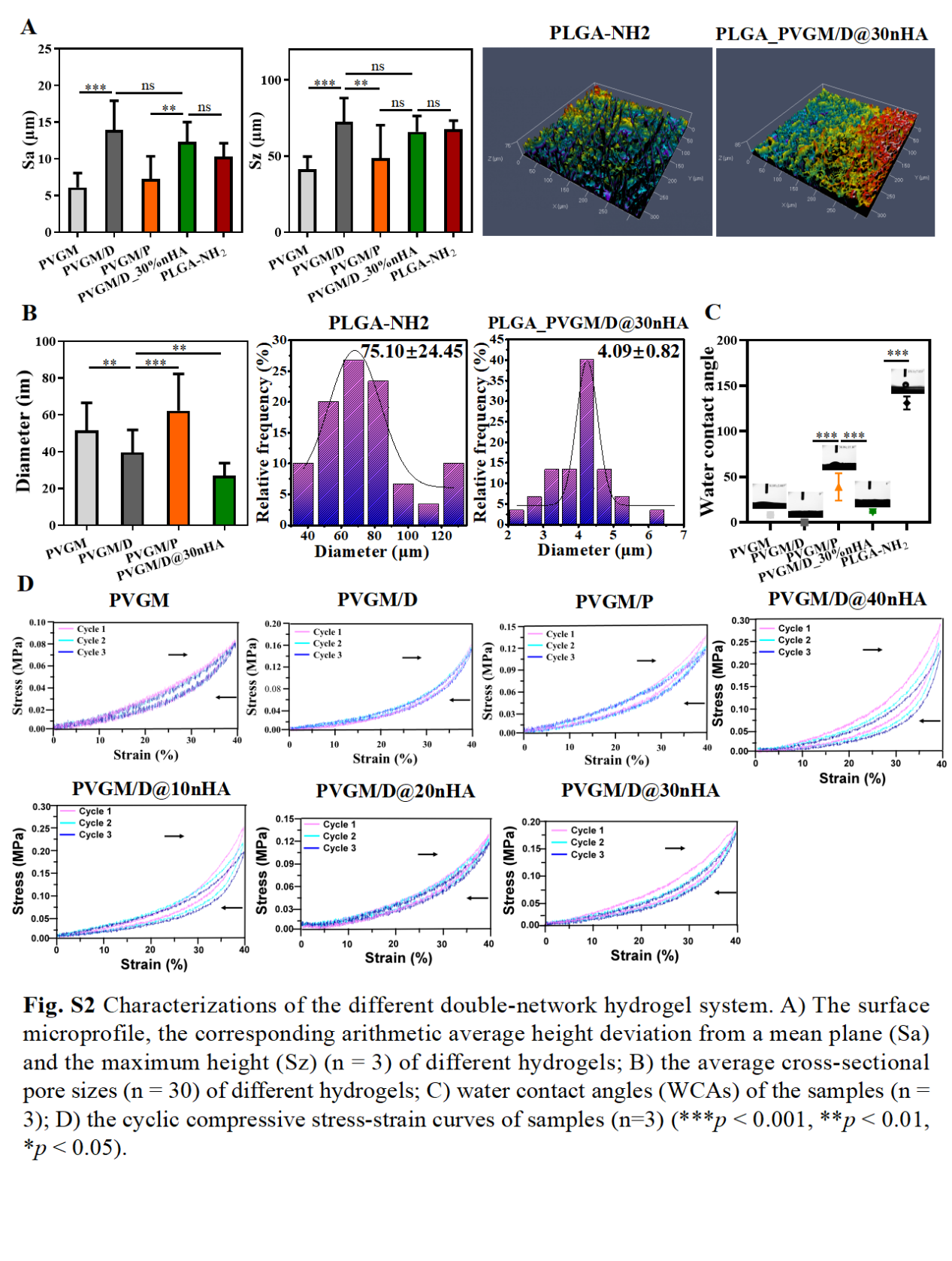


**Fig. S2** Characterizations of the different double-network hydrogel system. A) The surface microprofile, the corresponding arithmetic average height deviation from a mean plane (Sa) and the maximum height (Sz) (n = 3) of different hydrogels; B) the average cross-sectional pore sizes (n = 30) of different hydrogels; C) water contact angles (WCAs) of the samples (n = 3); D) the cyclic compressive stress-strain curves of samples (n=3) (****p* < 0.001, ***p* < 0.01, **p* < 0.05).

**
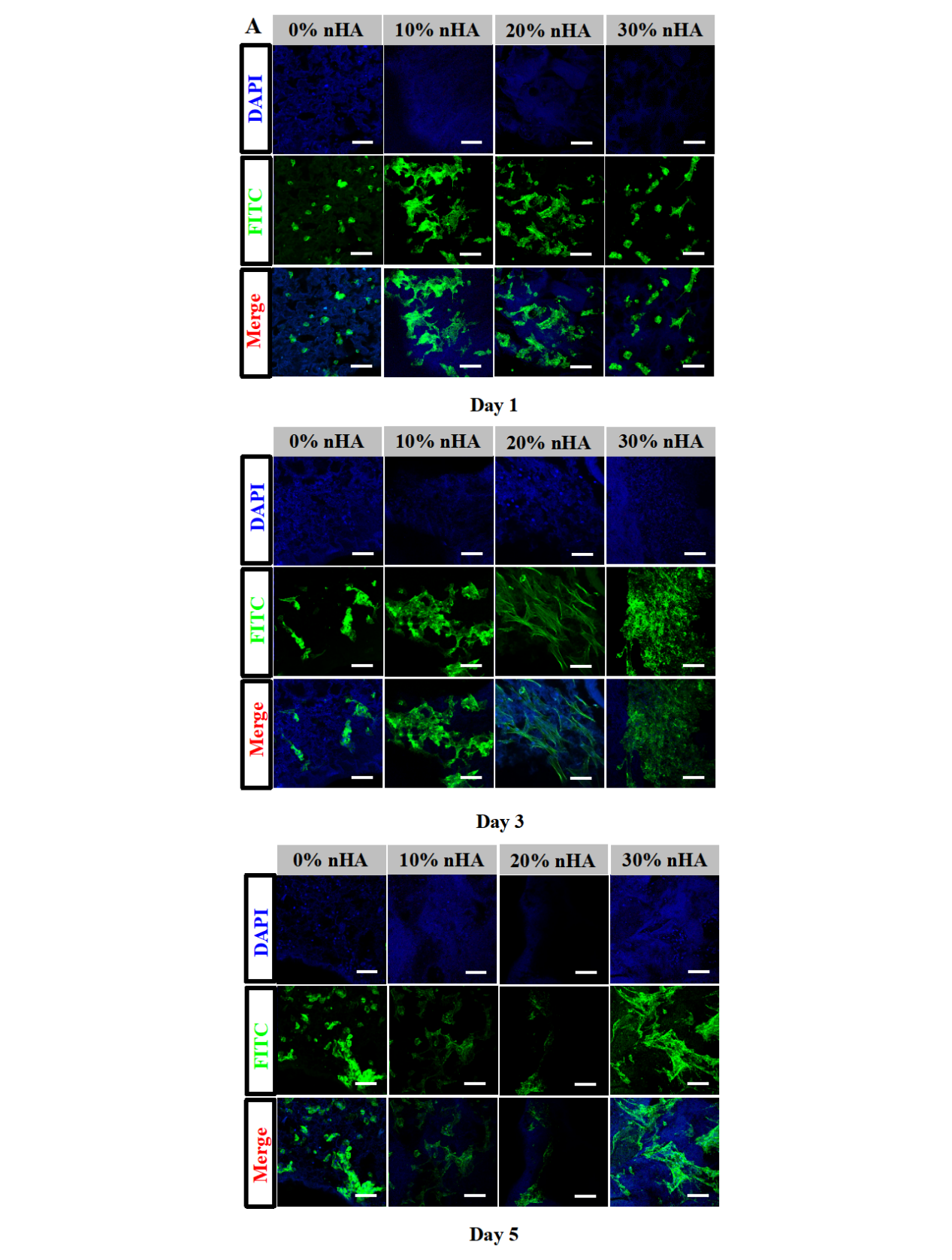
**

**Fig. S3** The BMSCs morphology after cultured on different hydrogels for 1, 3 and 5 days. The scale bar is 100 μm.

**
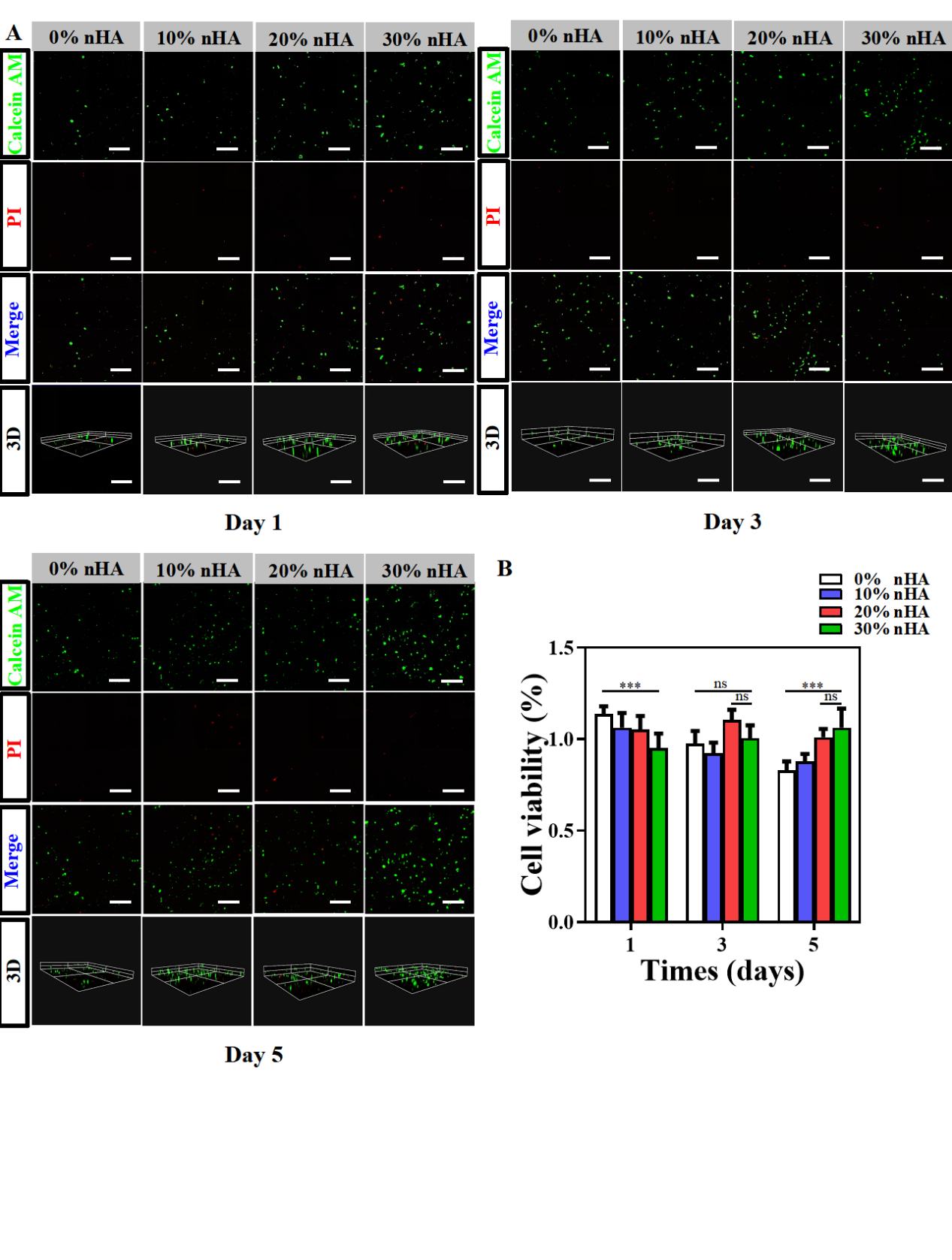
**

**Fig. S4** The cck8, live/dead staining and 3D reconstruction of BMSCs cultured for 1, 3 and 5 days. The scale bar is 200 μm.
.

**Table S1**. Contents of surface elements

| **Sample** | **Carbon（%）** | **Nitrogen（%）** | **Oxygen（%）** | **Calcium（%）** | **Phosphorus（%）** | **Boron (%)** |
| --- | --- | --- | --- | --- | --- | --- |
| PVGM | 49.86 | 17.44 | 32.69 | - | - | - |
| PVGM/D | 50.97 | 12.31 | 36.72 |  |  |  |
| PVGM/P | 53.48 | 7.00 | 26.92 | - | - | 8.66 |
| PVGM/D_30% nHA | 54.09 | 9.47 | 31.54 | 0.50 | 4.40 | - |
| PLGA-NH2 | 51.01 | 11.18 | 37.81 | - | - | - |

**Table S2**. The pore sizes of the hydrogel scaffold and the fiber sizes of hydrophobic layer

| **Sample** | **Minimum (μm)** | **Maximum (μm)** | **Mean (μm)** |
| --- | --- | --- | --- |
| PVGM | 24.48 | 84.27 | 51.64±14.94 |
| PVGM/D | 19.42 | 67.88 | 39.61±12.19 |
| PVGM/P | 31.60 | 93.78 | 62.29±19.99 |
| PVGM/D_30% nHA | 13.62 | 37.91 | 26.87±6.91 |
| PLGA-NH2 | 2.48 | 6.14 | 4.809±0.82 |

**Table S3**. The visualizations of the surface roughness

| **Sample** | **Sa (μm)** | **Sz (μm)** |
| --- | --- | --- |
| PVGM | 6.082±1.980 | 41.44±8.267 |
| PVGM/D | 13.92±3.992 | 72.48±15.70 |
| PVGM/P | 7.233±3.120 | 48.52±21.87 |
| PVGM/D_30% nHA | 12.33±2.665 | 65.95±10.43 |
| PLGA-NH2 | 10.32±1.815 | 67.57±5.826 |

**Table S4**. Primer sequences used for PCR measurements

| Gene | Primer sequence (F, forward; R, reverse) |
| --- | --- |
| GAPDH | F: CCGCATCTTCTTGTGCAGTG |
|  | R: ATCCGTTCACACCGACCTTC |
| ALP | F: GCCTACTTGTGTGGCGTGAA |
|  | R: AGGATGGACGTGACCTCGTT |
| RUNX2 | F: TCCGCCACCACTCACTACCAC |
|  | R: GGAACTGATAGGACGCTGACGAAG |
| OPN | F: CCAAGCGTGGAAACACACAGCC |
|  | R: GGCTTTGGAACTCGCCTGACTG |
| OCN | F: GCCCTGACTGCATTCTGCCTCT |
|  | R: TCACCACCTTACTGCCCTCCTG |
